# Effects of the Latex of *Synadenium grantii* Hook F. (*Euphorbiaceae*) on a Preclinical Model of Canine Prostate Cancer

**DOI:** 10.3389/fvets.2021.605286

**Published:** 2021-04-12

**Authors:** Eric Saymom Andrade Brito, Laís Di Paulie Taborda Prado, Liana Késia Costa Araújo, Emmanuel Arnhold, Moema Pacheco Chediak Matos, Joelma Abadia Marciano de Paula, Luciana Machado Ramos, Carlos Eduardo Fonseca-Alves, Veridiana Maria Brianezi Dignani de Moura

**Affiliations:** ^1^Animal Pathology Service, Federal University of Goiás- UFG, Goiânia, Brazil; ^2^Department of Animal Science, Federal University of Goiás UFG, Goiânia, Brazil; ^3^Unit of Exact and Technologic Sciences, Goiás State University, Anápolis, Brazil; ^4^Department of Veterinary Surgery and Animal Reproduction, São Paulo State University-UNESP, Botucatu, Brazil; ^5^Institute of Health Sciences, University of São Paulo—UNIP, Bauru, Brazil

**Keywords:** dog, cell culture, prostate cancer model, janauba, neoplasia, prostate

## Abstract

Prostatic cancer (PC) stands out in terms of its occurrence, pathophysiology, and unfavorable prognostics in humans and dogs. Natural drugs bear an integrative potential for conventional antineoplastic treatments. In this context, the bioproducts of *Synadenium grantii* have been empirically used in different parts of Brazil for the integrative treatment of prostate cancer in humans. However, there is no availability of scientific evidence of the antitumor effects of *S. grantii*. Therefore, this study aimed to investigate the bioactive compounds in the latex of *S. grantii* using the high-resolution mass spectrophotometry (HRMS) and to evaluate its cytotoxic effects on primary canine PC cell cultures. Four fragments of phorbol ester were identified as potential bioactive compounds using the HRMS. With the help of an MTT ([3-(4,5-dimethyldiazol-2-yl)-2,5 diphenyltetrazolium bromide]) assay, two canine prostatic carcinoma cell lines (PC 1 and PC2) showed a decrease in the tumor cell count, with an Inhibitory concentration 50 (IC_50_)of 0.8469 and 0.6068 mg/ml, respectively, for PC1 and PC2. In conclusion, the latex of *S. grantii* contains phorbol esters in its composition, and its aqueous solution has a cytotoxic effect on canine metastatic PC cells *in vitro*.

## Introduction

Prostate cancer (PC) is one of the most important problems worldwide. Most recent statistical report in the USA demonstrated that PC is the most common cancer among men excluding skin cancer. The 2020 cancer statistics also highlighted that one of five men may develop PC cancer (1). Recent release of the Global Cancer Observatory (2) pointed out that PC is one of the main leading causes of malign neoplasia-related cancer deaths in humans next to lung cancer and female breast cancer. It is the second most frequently occurring cancer among males and its occurrence has grown vertiginously throughout the globe (2). Due to its importance, several models, including PC spontaneous models, have been proposed (3). Dogs and humans are the only species in which PC occurs spontaneously, thus spontaneous canine PC has been used in comparative studies (3, 4) Canine PC has a low incidence but is characterized by its aggressive behavior, metastatic at diagnosis and poor outcome (5, 6). In addition, it is usually unresponsive to the androgen stimulation due to a lack of androgen receptor (AR) (3). Thus, dogs can be considered as an important model for the human androgen-independent PC (4, 7). Although dogs can be considered as a model, the veterinary literature is focused on comparative morphological characters, and the information on the molecular pattern of canine PC is limited (3, 4). Moreover, a very limited number of studies have investigated the antitumor effect of different compounds on the preclinical models of canine PC (8, 9). Thus, the evaluation of new potential drugs for preclinical models can benefit both dogs and humans.

Usually, anticancer drugs used in the conventional chemotherapy are originated from natural compounds, such as vinca alkaloids (10). Currently, 60% of the drugs for the anticancer therapy are sourced naturally, including from plants, microorganisms, and marine organisms (11). Selective and effective treatments, as well as new mechanisms to limit the illness progression, have been the goal of researchers for the development of anticancer agents (12). Thus, the plant-derived products are the sources of substances used in chemotherapy and other drug therapies (11, 13). A large number of natural active agents are present in cancer therapies (14, 15) because of their structural models and unique action mechanisms. The drugs obtained from natural products can be used as the drugs of primary chemotherapy (16), chemopreventives (17), or chemosensitizers, with the effects synergistic to the conventional chemotherapy (18). However, before using these compounds in clinical practice, the antitumor response and toxicity should be evaluated by *in vitro* models.

Brazil has a great natural plant biodiversity with a great potential to identify natural products with therapeutic properties (19). Most of the used plants are based on popular medicine, where people describe plant potentials to heal diseases without any scientific evidence (11). Among those plants, *Synadenium grantii* Hook F. (Euphorbiaceae) has been proposed to have an antitumor effect on human PC. The latex of *S. grantii* has been empirically used to treat allergic and gastric disorders and cancer (20). It is commonly administered orally in a solution diluted in water to treat malignant neoplasia. The latex of *S. grantii* is rich in non-polar substances (21, 22) and its activity against tumor cell lines has already been demonstrated (23–25). However, there is no finding of clinical evidence on the effect of the latex of *S. grantii* on patients with cancer. Based on this scenario, new studies are required for investigating such an effect on cancer cells. As dogs are considered as a model for human PC, the evaluation of the latex of *S. grantii* in canine cancer cell lines can be considered as a preclinical model, allowing future clinical studies in dogs. Unfortunately, no standard treatment has been tested on PC-affected dogs. Instead, chemotherapy with doxorubicin or carboplatin associated with non-steroidal anti-inflammatory drugs are commonly used (26). Thus, investigating new compounds for preclinical models can provide a new perspective. Therefore, this study aimed to investigate the bioactive compounds of the latex of *S. grantii* and evaluate its effects on canine PC cells as a preclinical model of canine PC.

## Methods

### Ethics Statement

This research was approved by the Research Ethics Committee of the Federal University of Goiás (CEP/UFG, protocol no. 2.269.572) and by the Ethics Commission for Animal Use of the São Paulo State University Júlio de Mesquita Filho (CEUA/UNESP, protocol no. 0004/2017).

### Plant Material

A fresh sample of the latex of *S. grantii* was collected in July 2018, in Nerópolis, Goiás, Brazil (16°24′31.8″ S, 49°13′11.3″ W, 812-m altitude) after cleaning and transverse cutting of the plant stem. The sample was acclimatized in a 50-ml Falcon tube and kept at 4°C. An exsiccate was prepared and deposited at the Herbarium of the UFG, under number 65903. Portion of the sample was used in the physicochemical characterization test and in the identification of active compounds. For *in vitro* and *in vivo* studies, 1 g of latex was dissolved in distilled water into a 10 ml final stock solution according to Luz et al. (27). Then, a decrease in concentrations was made, and new stock solutions with concentrations of 0.75, 1, 1.5, 3, and 6 mg/ml were originated.

### Physicochemical Characterization of the Latex of *S. grantii* Latex

Relative density of the latex was determined by a pycnometer method, weighing 10 ml pure latex in an AG200 analytical scale (Gehaka^®^, AG 200). The method was performed according to AGÊNCIA NACIONAL DE VIGILÂNCIA SANITÁRIA (28). Briefly, the relative density was the ratio of pure latex sample and distilled water weights. The same 10-ml sample was used in the other assays, including pH and humidity measurements, as well as the screening of phenolic and alkaloid compounds.

The pH was obtained by using a pH microprocessor (Marconi^®^, MA 522). Humidity was determined in the three 1-g latex samples by a desiccation loss analysis, using a moisture analyzer (Shimadzu^®^, MOC63u). For the detection of phenolic compounds, 1 ml of latex diluted in 10 ml distilled water was used, with the solution being heated for 5 min. Then, four drops of 4.5% ferric chloride were added, with hydroxyls determined qualitatively by evaluating dark precipitate in the solution.

Alkaloids were detected by diluting 2 g of the latex in 20 ml of 5% sulfuric acid. The solution was heated for 3 min, and drops of iodized and complex polyacids reagents were added to independent samples. Then, Mayer's reactive (potassium tetra-iodine mercurate), Dragendorf reactive (potassium iodine-bismuthate), Bertrand reactive (1% silico-tungstic acid), Hager reactive (2% picric acid), and 1% tannic acid were used.

### *S. grantii* Latex Phytochemical Research by High-Resolution Mass Spectrometry

A latex sample was subjected to the HRMS to identify 3,4,12,13-tetraacetylphorbol-20-phenylacetate, a compound previously isolated from *S. grantii* plants (29). Another sample was used to check whether the latex contain 12-deoxyphorbol 13-phenylacetate 20-acetate (dPPA). Lyophilized standards of both compounds were used for a comparison with the latex samples.

High-resolution mass spectrometry of latex samples and 3,4,12,13-tetraacetylphorbol-20-phenylacetate and dPPA standards were acquired by direct infusions, using the Q-Exactive (Thermo Fisher Scientific, Waltham, MA, USA) mass spectrometer, with the H-ESI (Thermo Fisher Scientific, Waltham, MA, USA) as a source. The equipment was operated in a positive mode under the following conditions: full scan (m/z 200–800), spray voltage of 3.5 kV, resolution of 70,000, 10 μl/min flow, sheath gas at 15 (arbitrary units), auxiliary gas at 2 (arbitrary units), capillary temperature of 32°C, auxiliary gas temperature of 37°C, and tube lens at 50 (arbitrary units).

Latex and standard samples were prepared in different ways. About 500 μl methanol and water (1:1) were added to a 0.5-ml *S. grantii* latex sample, and the solution was then subjected to an ultrasound for 3 min, filtrated, and acidified with formic acid.The standard sample of phorbol esters was prepared with methanol solvent and was prepared at a concentration of 10 ppm without acidification. The results were analyzed by using the Therm Platform, which allowed viewing of the total ion chromatogram at a range of 200–800 m/z. The compounds were evaluated by using a latex sample and phorbol ester standard fragments subjected to an impact analysis by electrospray mass spectrometry. Used solvent, mass number, carbons and unsaturation, Na+ and H+ gains, and H+ loss were all considered. A “blank” was performed to confirm whether processed ions were obtained from the sample or the solvent.

### Canine Prostate Cell Culture

Two canine prostate cancer cells lines (PC1 and PC2) were used in this study. PC1 was established from a 10-year-old, intact, mixed breed dog with non-metastatic PC (Gleason 10) and PC2 from an 11-year-old, intact, poodle dog with metastatic PC (Gleason 10). Cell morphology and phenotype were previously determined with both cell lines being negative for the nuclear AR expression, positive for prostatic-specific antigen (PSA) and pan-cytokeratin (AE1/AE3), and negative for uroplakin III, vimentin, and P63 (30).

PC1 and PC2 were used at passage 15 (P15) and cultured in a PrEBM medium (Lonza, Basel, Switzerland), supplemented with 10% fetal bovine serum (FBS) (Invitrogen, Carlsbad, CA, USA), with 1% penicillin-streptomycin solution (Sigma, Portland, OR, USA), in a humid atmosphere at 37°C and CO_2_ at 5%. To confirm the cell phenotype, PSA and AR immunofluorescence analyses were performed according to Costa et al. (30). Briefly, sterile circular coverslips (15 mm, Knitell) were placed at the center of each plate well with 12 sterile wells. A total of 1 × 10^5^ cells were sown in 250 μl of PrEBM medium (Lonza, Basel, Switzerland), supplemented with 10% FBS and incubated in a moist chamber at 5% CO_2_ and 37°C. When adherent cells reached 50% confluence, the medium was removed, and the cells were washed thrice with PBS and fixated with chilled methanol (4°C) for 15 min, followed by a permeabilization with the anionic surfactant solution Triton-X 0.25% (Sigma, Portland, OR, USA). The cells were incubated for 45 min with FBS at 3% in PBS and then used as a blocking solution. Afterward, they were incubated with rabbit polyclonal anti-PSA (Biorbyt, Cambridge, UK) and anti-rabbit polyclonal AR (1:50) (Santa Cruz Biotechnology, Santa Cruz, CA, USA). Finally, the cells were incubated by using Alexa Fluor 484 (Invitrogen, Carlsbad, CA, USA) diluted at 2 μg/ml. The slides were counterstained with 4′-6-diamidino-2-phenylindole (DAPI; Sigma, Portland, OR, USA). The coverslips were analyzed by using a confocal laser scanning microscope TCS SP 5 (Leica), and the images were captured by using the Leica Application Suite Advanced Fluorescence (LAS AF) software.

### Treatment and Cell Viability of the Latex of *S. grantii*

PC1 and PC2 cells were cultured for 24 h in 96-well plates, at 1 × 10^4^ cells/well, in a humidified incubator at 37°C and 5% CO_2_. Treatments of the latex of *S. grantii* were performed by using the samples of 0.75, 1, 1.5, 3, and 6 mg/ml, which were evaluated at 24 (G24), 48 (G48), and 72 h (G72). Negative control and blank groups were preformed according to Chaves et al. (31). After treatments, the media were discarded, and 10-μl MTT [3-[4,5-dimethylthiazol-2-l]-2,5-diphenyltetrazolium bromide, Sigma Aldrich, Portland, OR, USA] solution was used while maintained for 3 h in an incubator according to the manufacturer's recommendation. Thereafter, 100-μl solubilizing solution was added to each well. The plate was homogenized for 10 min, and optical density readings were performed in a microplate reader (Expert Plus, 595 nm). We determined the concentration at which 50% of the cell viability is inhibited [Inhibitory concentration 50 (IC_50_)] (32). Three independent experiments were performed thrice.

### Statistical Analysis

Descriptive statistics was performed, which describe qualitative outcomes as a positive and negative expression for each marker. For the IC_50_ determination, a dose-response curve was performed by using the GraphPad Prism v.8.1.0 (GraphPad Software Inc., La Jolla, CA, USA), at 5% significance level (*p* < 0.05).

## Results

### Characterization of *S. grantii*

The physicochemical characterization showed that the latex had a density of 1.02 g/ml and pH of 5.72.The percentage of total solids was 29.19% with 70.8% humidity. No precipitation occurred or change in coloration was noted in the sample used for a qualitative detection of phenolic hydroxyls. All reagents showed a discreet precipitation in the detection of alkaloid, which confirmed their presence in *S. grantii* latex samples. The standards used in the HRMS were standardized according to a technique wherein the molecular ion corresponds to its respective molecular mass. No specific molecular ion fragments characterizing the 12-deoxifolia-13-phenylacetate-20-acetate (dPPA), with m/z = 531.23 [M+Na]+, or the 3,4,12,13-tetraacetylforbol-20-phenylacetyl, with m/z 675. 27[M+Na]+z were found in the *S. grantii* latex.

**Figure 1:**
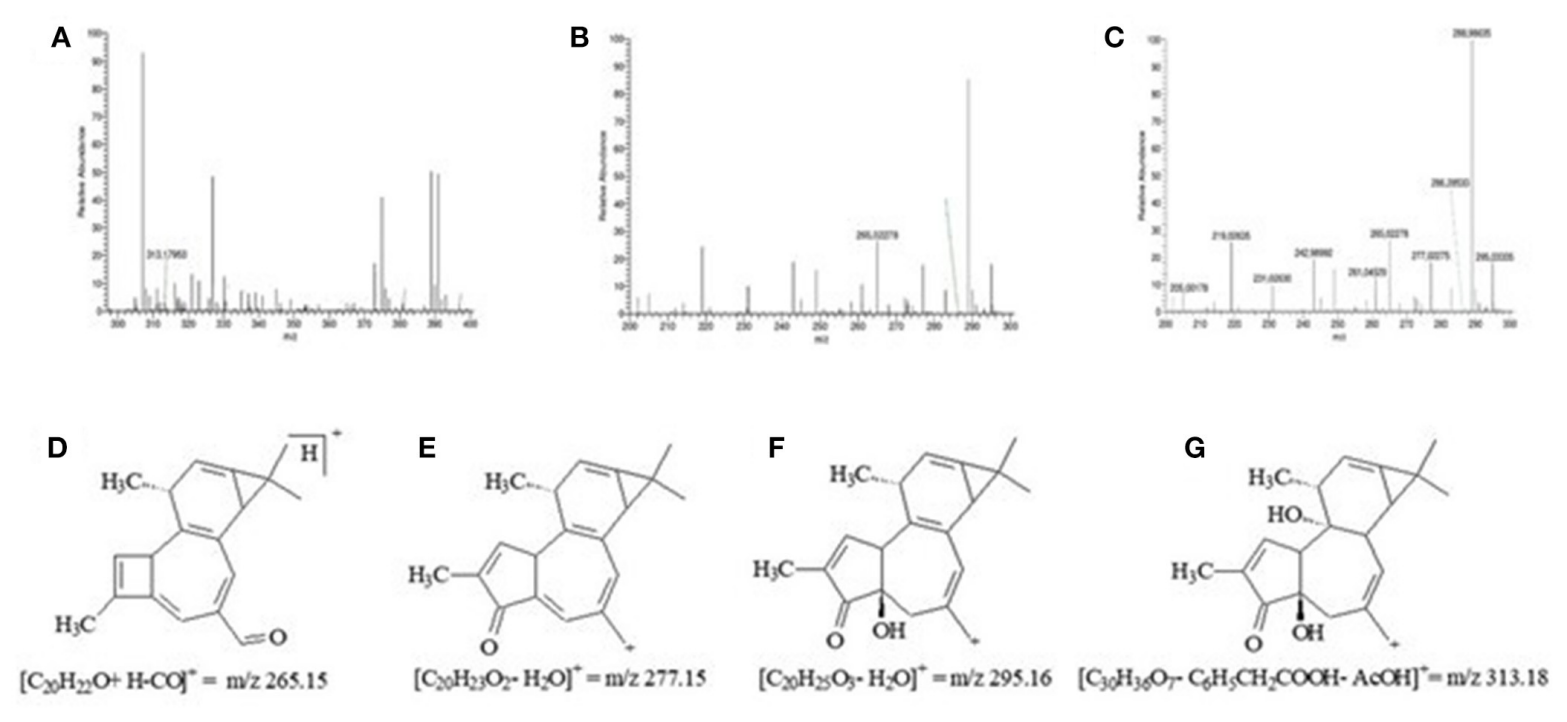
Peak generated in the mass spectrum (HRMS) of the latex of *Synadenium grantii* by magnifying the general chromatogram and its respective structures, molecular forms, and fragment masses. Chromatograms: **(A)** m/z 313.17; **(B)** m/z 265.02; **(C)** m/z 277.02 and 295.03. Structure, molecular form, and fragment mass: **(D)** m/z 265.02; **(E)** m/z 277.02; **(F)** m/z 295.03; and **(G)** m/z 313.17.

The compounds undergoing a test were not observed in the chromatogram of the latex of *S. grantii*. However, the HRMS analysis showed precisely the molecular weight of fragments of other compounds in the tested latex, including [C_20_H_22_O+H-CO]+ m/z 265.15, [C_20_H_23_O_2_-H_2_O]+ m/z 277.15, [C_20_H_25_O_3_-H_2_O]+ m/z 295.16, and [C_30_H_36_O_7_-C_6_H_5_CH_2_-COOH-AcOH ]+ m/z 313.18 (Figure 1), which are all from the phorbol family.

### Cell Phenotype and Viability After the Treatment of Latex of *S. grantii*

**Figure 2:**
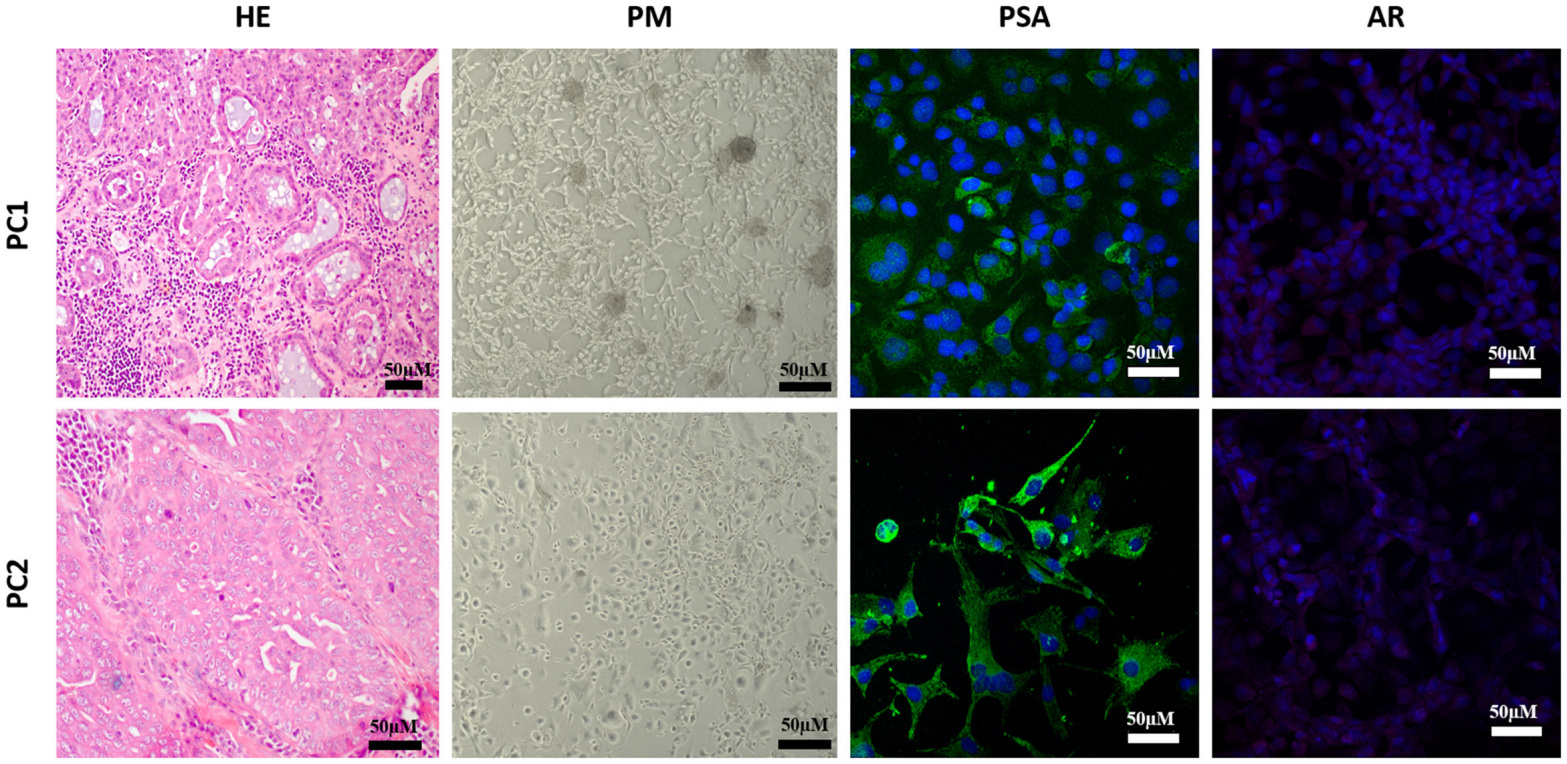
Morphological evaluation of two canine prostatic cell lines (PC1 and PC2). Both originated from Gleason 10 PC, with cribriform to solid morphology. In culture conditions, PC1 grew in groups, forming colonies, and tubular-like structures while PC2 grew isolated. The prostatic origin was confirmed by a prostatic-specific antigen (PSA) positive expression, and both were negative for androgen receptor (AR) expression. HE, hematoxylin and eosin; PC, phase contrast.

PC1 cells showed a polygonal morphology at P15 and grew in small clusters, forming cell colonies. PC2 cells also showed a polygonal morphology but grew isolated in the culture flask. In P15, cells are positive and negative, respectively, for PSA for RA (Figure 2).

**Figure 3:**
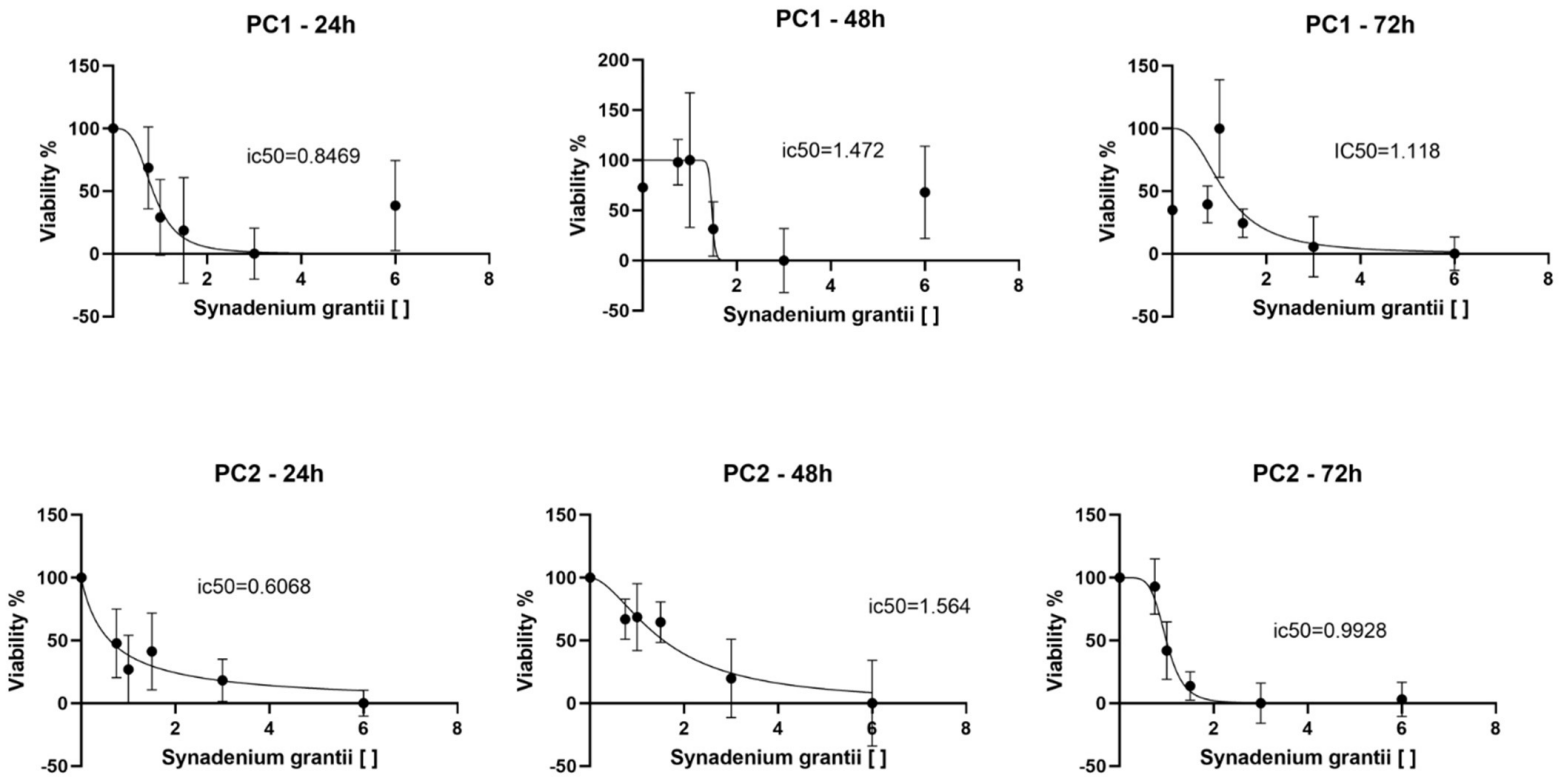
Determination of IC_50_ of PC1 and PC2 cell lines at 24, 48, and 72 h. The cell lines presented low IC_50_ at 24 h but high at 48 h. Then, at 72 h, IC_50_ decreased compared to the previous moment. The graphic representation also shows a dose-dependent response trend over time.

We identified different IC_50_ concentrations as a function of exposure time. Overall, after 24 h exposure, PC1 had an IC_50_ of 0.8469 mg/ml and PC2 0.6068 mg/ml (Figure 3). Both cell lines had high IC_50_ at 48 h but low at 72 h (Figure 3). All tested *S. grantii* latex concentrations had a dose-dependent effect on PC1 cells. When comparing 3 and 6 mg/ml with the untreated group, considering PC1 and PC2 within 24 h, 6 mg/ml had the best effects (0.19 and 20 abs), followed by 3 mg/ml in PC2 (0.27 abs). But within 72 h, cell viability had no differences in either PC1 or PC2 for both latex concentrations (Table 1).

**Table 1:** Mean cell viability for canine PC1 and PC2 cell lineages per concentration and exposure time to the latex of *S. grantii* by a tetrazolium reduction method.

	**PC1**		**PC2**	
	**Exposure time**			
**Concentrations**	**24 h**	**72 h**	**24 h**	**72 h**
0	0.24 ± 0.02^a^^*^^, A^^**^	0.48 ± 0.05^a,B^	0.56 ± 0.11^a,A^	0.48 ± 0.05^a,B^
3 mg/ml	0.26 ± 0.02^c,A^	0.21 ± 0.02^c,A^	0.27 ± 0.02^b,A^	0.21 ± 0.02^b,A^
6 mg/ml	0.19 ± 0.02^d,A^	0.21 ± 0.02^c,A^	0.20 ± 0.02^c,A^	0.21 ± 0.02^b,A^

*Scott-Knott test (p < 0.05%)*.

* lowercase letters in the same column are the comparing concentrations within the same time and

** *uppercase letters in the same row are the comparing concentration over different treatment times*.

## Discussion

Brazil has a great biodiversity with a potential to identify several compounds with antitumor activity based on native plants. Using technological databases, botanical taxonomists have classified plants into several kinds. In addition, advances in research have led to a finding of medicinal plants with a similar phenotype but differences in microscopic or molecular scales. Synonymy is quite common and Brazil counts with a species list, the “Lista de Espécies da Flora do Brasil” (33), which is an online list of accepted names of native species and their geographic distribution.

*S. grantii* is a species of the Euphorbiaceae family, also known as “milagrosa” or “janauba,” has been a scientific subject for the anticancer drug treatment since different compounds can be found in its plant material. Scientific evidence has found the anticancer activity of steroids, terpenes (diterpenes, sesquiterpenes, and triterpenes), phenolic compounds, and proteins from *S. grantii* (25, 27, 34, 35). Taxon evidence shows that *Euphorbia umbellata* (Pax) Bruyns and *Synadenium umbellatum* are scientific synonyms, as well as their role in anticancer drug development (27).

The use of the latex of *S. grantii* use as an antiproliferative agent has been experimentally demonstrated in several cancer cell culture studies. Other *Euphorbia* genera have been used in melanoma cell culture (25) and leukemic cell culture (36) for antiproliferative cancer research (37). In popular beliefs, the latex of *S. grantii* has a high antitumor effect when taking orally and is often advised for men with prostate cancer. However, no previous studies have demonstrated such an antitumor effect. Different *in vitro* and *in vivo* models have been used in a comparative oncology for preclinical research. For example, since prostate carcinoma affects both humans and dogs, the canine model has been widely accepted as a preclinical model for prostate cancer (38, 39). In addition, in recent years, several cultures of canine prostate cancer have been published, which allow testing of new drugs still in the preclinical phase (40).

Given the recent studies still on the latex of *S. grantii*, the first stage of our research was to characterize this latex. Previously, the density of this latex was reported to be of 1 g/ml when collected from the plant stem (41), which is close to water density and similar to what we observed. Total dissolved solids after the evaporation were not found for the latex in the literature. This parameter is useful in measuring working solutions since the water concentration in the latex must be accounted.

Plant primary and secondary metabolites are useful in determining the bioactive compounds for cancer research (42). Phenolic compounds are a group of substances with different levels of chemical complexity and are able to neutralize reactive oxygen species (ROS) (43). However, no phenolic compounds were detected in the samples of latex of *S. grantii* by using a qualitative analysis.

Cell morphology was analyzed to verify cell stability and confirm their phenotypes in cultures. Both cell cultures were previously characterized (30). However, we opted to confirm the AR status of cells and the cells being primary prostatic (based on PSA expression). In our study, more than 90% of the cells in both cell lineages of the canine PC-expressed PSA, which confirmed the prostatic origin of neoplastic cells. The role of androgens and the impact of early or late neutering are still controversial even though a few studies have not reported androgen effects on the canine prostate adenocarcinoma initiation or progression (6, 44, 45). In our study, AR immunoexpression was negative demonstrating androgen hormone independence for both cell lines.

We determined the IC_50_ for both cell lines and observed a dose-dependent response; therefore, the latex of *S. grantii* has antitumor activity in canine PC cells. The cytotoxic effect of plants of the *Synadenium* genus has been demonstrated mainly by the evaluation of plant shoot extracts (46, 47), with a few reports about it in the latex. This, in turn, has been used empirically in therapies and may be potentially dangerous due to its high toxicity, cytotoxic effects, and mutagenic potential (dose-dependent) besides its low lethal dosage to mice (110–168 mg/kg) (41, 48).

Such scientific information allied to the IC_50_ value of PC 1 and 2 suggests a potential effective and safe dose to be applied in future analysis. Thus, this safe dose can be tested in *in vivo* evaluation by using mice as a scientific model. The latex possesses a lethal action on cells at the proliferative stage (S-G2/M), which had been observed when using 1.7–7 *ug* latex per well in a melanoma cell culture by flow cytometry (25). Interestingly, PC2 come from a metastatic canine PC and presented an IC_50_ lower than PC1. In general, our results suggested an antitumor effect of the latex of *S. grantii in vitro*, which is important for future clinical trials involving this plant material in dogs with PC. The CAM assay showed significant results by comparing IC_50_ in PC1 and PC2 cells whereas metastatic cell lineage had a lower IC_50_.

## Conclusion

The latex of *S. grantii* plants contains phorbol esters in its composition. Its aqueous solution has an *in vitro* cytotoxic effect on metastatic canine PC cells. Still, the latex of *S. grantii* should be further verified for other biological effects, such as bioactive compound identification and respective action mechanisms.

## Data Availability Statement

The original contributions presented in the study are include in the article/supplementary material, further inquiries can be directed to the corresponding author/s.

## Ethics Statement

The animal study was reviewed and approved by this research was approved by Research Ethics Committee of the Federal University of Goiás (CEP/UFG, protocol no. 2.269.572) and by the Ethics Commission for the Use of Animals of the São Paulo State University Júlio de Mesquita Filho (CEUA/UNESP, protocol no. 0004/2017).

## Author Contributions

EB, LP, and LA were responsible for all experimental stages and for the writing of the first draft of the manuscript. EA was involved in statistical analysis. JP and LR were responsible for physicochemical and phytochemical characterization of the *S. grantii*. CF-A, MM, and VdM were responsible for the hypotheses, supervision of the *in vitro* and *in vivo* assays, as well as the writing and reviewing of the final draft of the manuscript. All authors contributed to the article and approved the submitted version.

## Conflict of Interest

The authors declare that the research was conducted in the absence of any commercial or financial relationships that could be construed as a potential conflict of interest.
